# Dollar store policy opportunities in Baltimore City: community member and policy maker perspectives

**DOI:** 10.3389/fnut.2024.1399402

**Published:** 2024-05-16

**Authors:** Samantha M. Sundermeir, Sydney R. Santos, Emma C. Lewis, Sara John, Karen Gardner, Emily Friedman, Lisa Poirier, Shuxian Hua, Sevetra Peoples-Brown, Sara E. Benjamin-Neelon, Joel Gittelsohn

**Affiliations:** ^1^Department of International Health, Johns Hopkins Bloomberg School of Public Health, Baltimore, MD, United States; ^2^The Center for Science in the Public Interest, Washington, DC, United States; ^3^Baltimore County Government, Towson, MD, United States; ^4^Office of Senator Mary Washington, Towson, MD, United States; ^5^Department of Health, Behavior, and Society, Johns Hopkins Bloomberg School of Public Health, Baltimore, MD, United States

**Keywords:** food access, food environment, community-informed policy, evidence-informed policy, Baltimore City, mixed-methods

## Abstract

**Introduction:**

There are currently over 50 dollar stores in Baltimore City, Maryland. Community perceptions of over-saturation and resulting neighborhood impacts have garnered recent attention. A Maryland State Senate Bill required further study of dollar stores in Baltimore City to inform future policy. Therefore, the over-arching goal of this study was to generate community-informed policy recommendations for the Baltimore City Council.

**Methods:**

Three methods of data collection were used: (1) in-depth interviews with community members, retail staff/owners, dollar store staff, and policy makers; (2) an online survey of Baltimore City residents; and (3) workshop with community members and one with policy makers. Triangulation across data sources, discussion amongst the research team, and member checking were used to generate the top four policy options: a conditional use ordinance, a community benefits agreements, a dispersal ordinance, and a staple foods ordinance.

**Results:**

There was strong support for policies that encourage dollar stores to better align with community priorities (e.g., improving store cleanliness and appearance, increasing availability of healthy foods), as opposed to closing or banning dollar stores entirely. There was also strong support for policies that would empower communities to participate in determining the role of dollar stores in their neighborhoods, for example through a conditional use ordinance or community benefits agreement. Key concerns included policy enforcement, given the additional funding required, and current limited capacity at the city government level. Strategies to address such challenges were generated including implementing business licenses at the city level, linking new ordinances to dollar store leases and permits, and encouraging dollar store participation in federal and local programs to more feasibly stock healthier food items (e.g., fresh produce). Dissatisfaction was expressed regarding a lack of policy options to address the existing dollar stores, not just new dollar stores entering the City.

**Discussion:**

This study is the first of its kind to assess community support for dollar store policies at the local level, and serves to inform policies that improve dollar stores. A report of these findings was provided to Baltimore City Council to inform new, community-supported dollar store policies.

## Introduction

With over 34,000 locations throughout the United States (US) (1), dollar stores have become a popular small box, discount retail establishment, particularly in small, rural towns and urban communities (2). Dollar stores in this context are operated by two corporations: Dollar General and Dollar Tree (which also owns Family Dollar) and sell a variety of “everyday” items from household and office supplies, apparel, to craft, party and seasonal products, to pre-packaged and refrigerated foods (3–5). They tend to be prevalent in some of the country’s most vulnerable, low-social economic status communities, filling in gaps where other retailers are absent (6, 7). In recent years, dollar stores have garnered increased attention from community members and policy makers across the country (8, 9). Their successful business model has allowed for rapid proliferation, but some concerns have been raised regarding density in certain areas (6, 10) and potential impact on other local businesses [such as independently owned food retailers (11, 12)], the lack of healthy food options available (9, 13), as well as reports of inadequate staffing (9) and poor treatment of staff (14). The cleanliness and upkeep of dollar stores has been a point of contention as well. Since 2017, the Occupational Safety and Health Administration has issued millions of dollars in fines to dollar store companies found in violation of safe and healthy working conditions (15).

According to the Institute for Local Self Reliance, since 2019, there have been at least 75 new dollar store proposals blocked throughout the US; additionally, at least 54 of those municipalities have taken policy action to address concerns and optimize the role of dollar stores in communities (1). Most policy actions stem from community-driven organizing efforts to limit dollar store proliferation and/or improve existing dollar store offerings and practices to better serve their community (9). To date, common policy mechanisms implemented to effect change include moratoriums (prohibits new dollar stores from opening for a specified time period), special exception/conditional use (requires local government approval to open a new dollar store), prohibited use (prohibits certain types of land uses for specific areas), and overlay districts (a district applied over an existing district or districts to establish new property standards) (8). A strong focus of these policies has been to encourage new dollar stores to offer healthier options.

There are currently over 50 dollar store establishments across the three brands (Dollar General, Dollar Tree and Family Dollar) throughout Baltimore City, Maryland, and this number is expected to continue rising. Similar to concerns raised by other communities across the country, some Baltimore City residents have expressed concerns of dollar store saturation to their state policy makers. In response, Maryland State legislators introduced Senate Bill (SB) 869 in 2022 to strengthen zoning requirements and establish community agreements as part of the City’s land use authority over small box discount stores. To address the concerns outlined and provisions proposed throughout SB 869, the Maryland State Delegation advised for the completion of a comprehensive study of dollar store establishments in Baltimore City. Part of this research included exploration of community member and policy maker support for dollar store policies at the local level, given that no previous studies have done so. Therefore, the over-arching goal of this study was to generate community-informed policy recommendations to present to the Baltimore City Council. The objectives of this study were to: (1) identify the top dollar store policy opportunities supported by Baltimore City community members and policy makers, and describe their perceived benefits and challenges; and (2) discuss strategies for addressing perceived challenges related to these dollar store policies.

## Methods

### Setting

Baltimore City is estimated to have nearly 570,000 residents; about two-thirds of the population identify as Black or African American (16). A higher proportion of Black individuals reside in a Healthy Food Priority Area of the City [defined as a neighborhood where the average Healthy Food Availability Index score is 9.5 or less out of 28.5, median household income is <185% of the Federal Poverty Level, >30% of households have no vehicle, and the distance to any supermarket is >0.25 miles (17)] compared to White individuals (31% vs. 9% respectively). Baltimore is comprised of many small food sources (e.g., corner stores, carryouts) but has proportionally fewer supermarkets (18).

### Study design and overview

This is a sub-analysis of a larger study of dollar stores in Baltimore City which employed a sequential mixed-methods approach (19) to understand the role of dollar stores in the food environment and the community. The study had three phases where each phase informed the next: (1) in-depth interviews, (2) in-store structured observations and an online survey of Baltimore residents, and (3) two workshops (one with community members and one with policy makers). Findings from the in-store observations are reported in a publicly available dollar store report (20).

### Inclusion criteria

To be eligible to participate in any phase of the project, participants had to be at least 18 years old and live or work (i.e., dollar store staff, retail food store staff, policy makers) in Baltimore City. For the community member workshop, participants that shopped at dollar stores at least once per month were prioritized in order to contribute knowledgably to the discussion. An initial analysis of the online survey data after receiving 91 responses revealed a largely food secure sample. As a result, the Hunger Vital Sign (21) food security screener was added as an additional screening question in order to diversify the sample by food security status.

### Data sources

The present study used three data sources from the larger study: in-depth interviews, the online survey, and the two workshops.

### Step 1: in-depth, semi-structured interviews

In-depth, semi-structured interviews were conducted with Baltimore City residents, current and former dollar store staff, retail staff/owners, and policy makers from December 2022–June 2023. Participants completed one, 45–60-min interview either via Zoom or in-person conducted by trained graduate research assistants in the Department of International Health. Interview guides were developed by the research team at the Johns Hopkins School of Public Health, and through discussions with community collaborators (i.e., partners at the Health Department and Department of Planning in Baltimore City). Participants were asked about their perceptions of and shopping habits at dollar stores in their neighborhoods, what an ‘ideal’ dollar store would look like to them, and their views on dollar store policies, including those passed elsewhere (e.g., dispersal ordinances, conditional use ordinances, healthy food stocking requirements). Policy makers were asked directly about potential policy options based on strategies successfully utilized in other locations and the needs expressed by community members (8). Interview debriefing occurred at weekly team meetings, and data collectors updated probing strategies based on new topics that arose. All interviews were recorded via Zoom or a digital recording device, and transcribed using the transcription feature in Microsoft Word. Research assistants cleaned each transcript and checked them for accuracy.

### Step 2: online survey

An online survey of Baltimore City residents was conducted using Qualtrics (22) from July–October 2023. The survey was adapted from the Center for Science in the Public Interest’s (CSPI) nationwide dollar store survey (9), and updated based on the information gathered from in-depth interviews. The survey included: (1) sociodemographic questions [age, race/ethnicity, sex, use of Supplemental Nutrition Assistance Program (SNAP), and food security status using the validated 2-item Hunger Vital Sign (21)], (2) questions about dollar store perceptions and shopping behaviors, and (3) support for dollar store policy features. Participants were asked to rank potential policy options from a list of policy features generated from in-depth interviews and examples from other municipalities. A Likert scale was used to indicate support for each policy feature, ranging from “strongly disagree” to “strongly agree”.

### Step 3: workshops

The study culminated with two in-person workshops: one with Baltimore City community members on July 31, 2023, followed by a second with city-level policy makers on August 3rd, 2023. The workshop scripts were developed and led by the research team and CSPI staff, and were informed by prior data collection. Workshops were recorded using digital recording devices, and transcribed using the transcription feature in Microsoft Word. Research assistants cleaned and checked each workshop transcript for accuracy.

Prior to the workshops, a draft dollar store policy was received by the research team from City Council which was slated to be introduced later in the year. Thus, the workshop guides were updated to include a presentation of the draft bill to discuss how to improve the bill and better align its contents with community priorities related to dollar stores.

The goal of the workshops was to present the findings from the previous phases of the dollar store study, and facilitate the co-generation of top policy recommendations for Baltimore City Council. The community member workshop started with each participant sharing one asset and one challenge in their neighborhood. Key words were recorded on a flipchart by research assistants. Then, participants voted on the most important challenges amongst those generated: the votes were tallied and the top three challenges were announced to the group. Next, to begin discussing dollar stores, participants were asked about how they thought dollar stores did or did not contribute to the assets and challenges in their neighborhoods. Key words/phrases generated were recorded on the flipchart. After that, a short PowerPoint presentation about the study findings from phases 1 and 2 were presented. The first half of the workshop was ended by displaying the top five policy options from the larger study on the screen for participants to consider during a 10-min break. The second half of the workshop focused on potential dollar store policies. First, reactions to the top policy options generated from the larger study were solicited. Examples from other municipalities were presented for each one as applicable, and specific questions about the details of each type of policy were answered by CSPI staff. Then, the draft City Council conditional use Bill was introduced. Participants were asked to discuss their initial reactions, perceived pros and cons, and strategies for improving the Bill based on community priorities discussed in the first half of the workshop. The ideas generated were recorded for incorporation in the subsequent policy maker workshop. The workshop lasted three hours and 15 min.

Three days later, the policy maker workshop took place at the Johns Hopkins Bloomberg School of Public Health. The workshop started with an overview of the study design and purpose, and a presentation about initial findings from the in-depth interviews, store observations, and the online survey. After discussing the findings and answering questions that came up, findings from the community member workshop conducted earlier in the week was presented to policymakers, including the outputs generated (top neighborhood challenges, policy support/concerns/suggestions). After a 10-min break, an overview of the proposed City Council conditional use Bill was presented. The research team facilitated discussion around how the policy could be improved to align with community priorities generated in the community member workshop. Probing questions included participants’ own views of the Bill, and any concerns they had. The workshop lasted two hours and 28 min.

### Recruitment

For in-depth interviews and workshops, two recruitment methods were used: (1) purposive sampling and snowball sampling to identify participants who were knowledgeable about dollar stores and/or food access in Baltimore and (2) convenience sampling using email listservs (through neighborhood associations, City Council, Department of Planning and the Baltimore City Food Policy and Action Coalition) and distributing/posting flyers in the community, and attending neighborhood association meetings and community events. Due to difficulties recruiting current and former dollar store staff, three former dollar store staff members from outside of Baltimore City were included in in-depth interviews.

Convenience sampling was used to recruit online survey participants via neighborhood association, Department of Planning, and the Baltimore City Food Policy and Action Coalition email listservs, and through distributing/posting flyers in the community (libraries, recreation centers, senior centers), and attending community events, especially within in zip codes where there were no responses at the time of the recruitment outing.

### Ethical considerations

This study was approved by the Johns Hopkins School of Public Health Institutional Review Board (IRB00022523). All participants completed informed consent prior to engaging in an interview, workshop, or online survey. Interviewees and survey respondents received electronic $20 Visa gift cards, and workshop participants received physical $100 Visa gift cards for participation. Policy makers were offered gift cards, but all refused to accept compensation.

The research team was comprised of predominantly White students and faculty and the Johns Hopkins Bloomberg School of Public Health. Policy expertise and technical support was provided by three staff members at CSPI. The research team acknowledges the biases this may have introduced to the research process. A mixed-methods approach contributed to the goal of obtaining multiple perspectives across diverse participant types. Best practices for creating survey questions, interview questions, and workshop guiding questions to avoid loaded and leading questions (23, 24).

## Analysis

The top policy options were identified based on thematic analysis, triangulation across data sources, and discussion amongst the research team. The top policy options selected were triangulated across data sources based on those that came up frequently and were emphasized the most, were considered to be most feasible by the participants, and based upon research team discussion and member checking with community partners. For qualitative data, a combination of deductive and inductive coding was used to generate an initial list of codes based on the policies identified, and the codebook was updated as needed during the coding process. The first four transcripts were coded by two coders (SMS and SS) who then met to discuss and adjust the codebook. The remaining transcripts were coded by the first author (SMS). Once coding was complete, thematic analysis was used to construct a matrix for each policy option which included evidence for being supportive or not for the policy, perceived benefits and challenges, and strategies for addressing challenges. All coding was completed using Dedoose version 9 (24). For quantitative data, policy rankings from the online survey were generated by adding up the number of respondents who indicated that they either “agreed” or “strongly agreed” with each policy feature presented. Quantitative analyses were conducted using Microsoft Excel.

## Results

### Overview

Table 1 provides an overview of study participant type and characteristics. Twenty-five people completed interviews (excluding two people who were excluded because they were not Baltimore residents). Six of the community member interviewees resided in a Healthy Food Priority Area of Baltimore [Healthy Food Priority Area replaced the term “food desert” in Baltimore City and are defined as are defined as neighborhoods where the average Healthy Food Availability Index score is 9.5 out of 28.5 or less, median household income is <185 of the Federal Poverty Level, >30% of households have no vehicle, and distance to supermarket is more than 0.25 miles (17)]. The final sample for the online survey was 120 participants across 21 zip codes where most (47%) identified as White, and (49%) were food insecure. Twenty-one people participated in the community member workshop where the majority (63%) identified as Black, 44% were food insecure. Eight participated in the policy maker workshop where (50%) identified as Black.

**Table 1:** Participant characteristics from each phase of the study of dollar stores in Baltimore City.

Study phase	*N*	Characteristic	*N* (%)
In-depth interviews	25	Participant type Community members Current dollar store staff Former dollar store staff Retail business owner Food retail staff Policy makers	13 1 3 1 2 5
Online survey	120	Race/ethnicity (*n* = 119) Asian Black Black and Hispanic/Latino White White and Hispanic/Latino White and Native American or Pacific Islander Declined to answer Age 18–24 25–34 35–44 45–54 55–64 65+ % Female Employment (*n* = 119) Full time Part time Self employed Not employed outside the home Retired Educational attainment (*n* = 119) Some high school High school graduate Vocational/two-year/community college Some four-year college Four-year college graduate Advanced degree Experiencing food insecurity (% yes) SNAP Participant (% yes) Dollar store shopping frequency Any dollar store shopping Frequent dollar store shopping^*^	2 (1.7) 51 (42.9) 1 (0.8) 56 (47.1) 1 (0.8) 1 (0.8) 7 (5.9) 13 (10.8) 33 (27.5) 21 (17.5) 21 (17.5) 18 (15.0) 14 (11.7) 79 (65.8) 80 (67.2) 14 (11.8) 9 (7.6) 4 (3.4) 12 (10.1) 3 (2.5) 22 (18.5) 10 (8.4) 12 (10.1) 33 (27.7) 39 (32.8) 59 (49.2) 31 (25.8) 57 (47.5) 38 (31.7)
Community member workshop	21^†^	Race/ethnicity (*n* = 19) Asian Black White Declined to answer Age (*n* = 19) 18–24 25–34 35–44 45–54 55–64 65+ % Female (*n* = 19) Educational attainment (*n* = 19) Some high school High school graduate Vocational/two-year/community college Some four-year college Four-year college graduate Advanced degree Experiencing food insecurity (% yes) (*n* = 18) Dollar store shopping frequency (*n* = 19) Daily A few times/week Once per week A few times/month Once per month or less Never	1 (5.3) 12 (63.2) 5 (26.3) 1 (5.3) 0 3 (15.8) 3 (15.8) 2 (10.5) 7 (36.8) 4 (21.1) 14 (73.7) 1 (5.3) 3 (15.8) 3 (15.8) 1 (5.3) 2 (10.5) 9 (47.4) 8 (44.4) 0 3 (15.8) 2 (10.5) 5 (26.3) 7 (36.8) 2 (10.5)
Policy maker workshop	8	Race/Ethnicity Asian Black White Hispanic/Latinx Declined to answer Age 18–24 25–34 35–44 45–54 55–64 65+ % Female Educational attainment Some four-year college Four-year college graduate Advanced degree Experiencing food insecurity (% Yes) Dollar store shopping frequency A few times/month Once per month or less Never	0 4 (50.0) 2 (25.0) 2 (25.0) 1 (12.5) 1 (12.5) 4 (50.0) 1 (12.5) 1 (12.5) 0 1 (12.5) 2 (25.0) 1 (12.5) 2 (25.0) 5 (62.5) 1 (12.5) 1 (12.5) 3 (37.5) 4 (50.0)

^†^There were 21 workshop participants. Two of them declined to complete the questionnaire leaving a total *N* of 19 for demographic analysis. ^*^Frequent shopping was defined as shopping at a dollar store more than once per month. SNAP, Supplemental Nutrition Assistance Program.

The ranking of policy features from the online survey are presented in Table 2. Among survey respondents, the highest-ranking policy features were those that improve the interior and exterior cleanliness and appearance of dollar stores and help attract other food retailers. The lowest ranking policy features were policies that close existing dollar stores and ban new dollar stores from opening.

**Table 2:** Policy feature rankings among online dollar store survey respondents in Baltimore City.

Do you agree or disagree with the following statements as it relates to your neighborhood? A new policy should…	% Agree* overall (*n* = 120)
Do more to improve the cleanliness and appearance inside of dollar stores	77.5%
Do more to improve the cleanliness and appearance on the outside of dollar stores	76.7%
Help attract other food retailers, besides dollar stores, that would be beneficial to my neighborhood	75.0%
Outline proper staffing and wages at dollar stores	74.2%
Require dollar stores to stock food items such as fresh fruits and vegetables, milk, eggs, dairy, fresh meat, and whole grains.	65.8
Support improved security at dollar stores	65.8%
Require dollar stores to source produce locally or regionally	60.8%
Only allow a dollar store to open if a portion of their floor space is dedicated to stocking healthy food items	57.5%
Require that the surrounding neighborhood be notified of a new dollar store opening	56.7%
Require approval from the surrounding neighborhood before a new dollar store can open	55.8%
Should require a new dollar store to be a certain distance away from an existing dollar store	55.0%
Allow existing dollar stores to stay open, but should not allow new dollar store to open	33.3%
Close existing dollar stores	22.5%

### The top community-supported dollar store policies

Table 3 summarizes the top policy options, along with perceived benefits and challenges for each.

**Table 3:** Top dollar store policies identified across data sources and their corresponding perceived benefits and challenges.

Policy	Definition	Perceived benefits	Perceived challenges
Conditional use	Local government approval required for land use approval. The approval process involves assessing alignment with conditions or standards outlined in an ordinance.	Requires certain conditions to be met before a new store dollar store can open, potentially limiting how many stores there are and avoiding further saturation. Allows more time for community members and city agencies to plan and respond to a new dollar store proposal.	Inequitable for communities with limited capacity and resources. Does not address existing dollar stores.
Community agreements	An agreement between a dollar store company, land or building owners, and the surrounding community about certain terms and conditions that must be met.	Empowers communities. Communities and dollar stores can work together to meet community needs.	Difficult to enforce the terms of agreement. Does not address existing dollar stores.
Dispersal ordinance	Requires new stores to locate a certain distance from any existing store.	Decreases dollar store saturation. Protects local small businesses.	May be viewed as discriminatory against dollar stores. Does not address existing dollar stores.
Staple foods ordinance	Requires new stores to stock certain healthy food items such as fresh produce, meat, dairy, and whole grains.	Improves healthy food access. Fills a need in communities with limited grocery and food options. Has the potential to address existing dollar stores.	Difficult to enforce: requires additional capacity at the city level.

#### Conditional use ordinance

Both community members and policy makers were in favor of a conditional use policy, which would require the local government to assess how each proposed dollar stores aligns with certain conditions or standards. For example, a conditional use policy could include language to require community approvals, community benefits agreements, and/or dispersal distances.

Community members viewed this mechanism as a tool they could use to help ensure new dollar stores (and other retailers entering the neighborhood) aligned with and supported their neighborhood strategic plans and visions. Many participants spoke about existing neighborhood plans, such as five- or ten-year plans, that guide neighborhood development. They often include plans for the design and appearance of the neighborhood, building design (e.g., brick buildings to match the existing historic aesthetic), and the types of retail and business they’d like to attract.

However, there was concern that a conditional use policy would not adequately serve neighborhoods with less capacity (e.g., less funding and manpower). If this were the case, neighborhoods without a neighborhood association, funds, or awareness of certain issues may not benefit from a conditional use policy. For example, one policy maker discussed that typically a conditional use application goes through unless it is opposed:

“*…conditional uses are generally presumed to be acceptable, unless a contester can show why it is not according to a set of required findings and considerations. That means that it’s generally presumed allowed, so somebody that does not want it has the burden of proof, as it were, to show why it’s problematic… They’re almost all volunteers that have normal lives and other obligations. Very few of them are experienced in land use anything, especially in neighborhoods with lower average educational attainment. Just understanding how the process works is a challenge for them. Whereas, our higher educationally attained neighborhoods that have a lot of retirees that have money to hire attorneys on their behalf. Yeah, it’s not a fair fight.”* – Policy maker

Participants also highlighted the concern that a conditional use ordinance would only address new dollar stores entering Baltimore, and does not address existing stores.

#### Community benefits agreement

Having a community benefits agreement in place can be included as a condition to be met under a conditional use ordinance. There was strong support for including a community benefits agreement requirement in a conditional use dollar store ordinance in Baltimore City. Participants felt that given the sheer number of dollar stores located in Baltimore City, they should also be encouraged to benefit the communities they serve. And, participants wanted a say in what those benefits should be, and highlighted that preferred benefits may differ by neighborhood or community. This sentiment aligns well with what’s feasible in terms of policy, given that a conditional use policy (to which the local government is not a party) cannot require certain terms within a community benefits agreement, but can suggest language. Therefore, a future conditional use ordinance in Baltimore could include suggested language for the agreement, which would be a good starting point for neighborhoods, especially those with limited resources. See Table 4 for community benefit agreement context ideas generated across in-depth interviews and workshops.

**Table 4:** Strategies for implementing community benefits agreements and a staple foods ordinance generated from in-depth interviews and workshops.

Community benefits agreement	Staple foods ordinance
Content related to store cleanliness and appearance inside and outside of the store Stocking of healthy foods Sourcing food from local vendors and producers Align the design of a new store with the look of the surrounding neighborhood (e.g., brick facades with windows of instead of windowless concrete facades) Staff-related content Hire locally (i.e., within store zip code) Fair wages Provide additional benefits (stock options, some form of ownership, healthcare stipends) Invest in the neighborhood Participate in local programs (e.g., the Fresh Crate program, which is a collaborative effort between a university and small business to bring produce into the Baltimore City (25); business partnerships in Baltimore who fund local initiatives for keeping the neighborhood clean and safe) Provide community grants and/or scholarships for schools in the area, Accessibility Improve accessibility for those who are physically or visually impaired so they can navigate the store more easily by keeping the store aisles clear and free of clutter	Work with local suppliers Some suppliers are already delivering produce to other places (like restaurants) in the city on a daily basis. This system could be leveraged to make stocking produce easier for dollar stores Provide incentives, such as small grants for refrigeration Encourage participation in local programs like Fresh Crate (25) Demonstrate to dollar stores that these items successfully sell in other store types around the City, like corner stores. Use the building code to require certain amount of food refrigeration The City could provide the building space, and dollar stores could lease the building from the City. In this approach, the City would take on the burden of maintaining the building, which lowers overhead costs for dollar stores Then, the City could require the refrigeration and stocking of healthy food items in order to get the lease Encourage dollar stores to take advantage of tax credit* They would qualify for existing tax credits if they had meat and produce sections Community members want to ensure the tax revenue goes back into their neighborhoods

* Some community members and policy makers were not in favor of allowing dollar stores to take advantage of these mechanisms, as they were meant to help attract full-service grocery stores, not dollar stores.

There were concerns amongst community members and policy makers about the legal weight of the content of a community benefits agreement. Community members worried that dollar stores would make empty promises in order to open a store without following through, with no clear enforcement strategy for the agreement. Policy makers also warned that adding too many requirements for stores to meet could deter business from entering a neighborhood, especially given existing challenges attracting new businesses; community members did not necessarily see this as a negative when asked what their thoughts were on this:

*“I think if people want to come in your community, they will go through the process like that, [they] go through the process for anywhere else or anything else. It’s all about dollars and cents. And they will go through whatever process they need to go through to make it happen. And if they realize that you do have a neighborhood that is aware and conscious of what’s going on and want to be involved, that may make them take better care of the property.”* – Community Member

The corporate structure of dollar store companies is a concern when it comes to getting them to agree to the terms of a community benefits agreement. One concern is that they will have responses ready for a lot of the requested terms for community agreements. For example, they might say they already do things like charitable donations or have written procedures for store internal and external store upkeep, when in practice this those actions may not directly benefit the community or be upheld.

Again, participants highlighted that a community benefits agreement would only address new dollar stores entering Baltimore, and does not address existing stores.

#### Dispersal ordinance

Dispersal ordinances require dollar stores to locate a certain distance from any existing dollar stores. There was support for a dollar store dispersal ordinance given that many participants recognized not only how many dollar stores already exist throughout the city, but also how concentrated they are in some areas. Support for this type of policy also stemmed from wanting to protect other businesses, especially “mom and pop” stores owned by locals and other food stores. Many discussed the lack of food stores in their neighborhoods and were concerned that dollar stores may keep businesses like grocery stores out, and consequently lead to negative implications for neighborhood health outcomes:

*“It has its place, but it should not be in close proximity and it should not be something that almost turns into a competitor with an actual grocery store, which is offering all of the foods. And to have them saturate the city the way that they are doing, they are literally going to drive your health costs up. They’re going to drive up.”* – Food Retail Staff

When asked what the best distance requirement would be, community members did not feel that they had enough information to decide. They were interested in knowing what distances other cities have used, and some simply said “the maximum distance possible”.

Policy makers were concerned that this policy could be viewed as discriminatory against one specific store type, given that it would only apply to dollar stores.

#### Staple foods ordinance

Community members and policy makers favored a staple foods ordinance, which would require dollar stores to maintain a minimum stock of certain healthy food items. This sentiment relates to the high density of dollar stores within City limits, and the desire to leverage them to serve communities well. Healthy food access is a big topic of interest in Baltimore City, and there are numerous individuals, groups, institutions, and neighborhoods working on creative, sustainable solutions to this issue (25–30). Participants did not want to shut down dollar stores, but instead preferred to improve them in part by improving the types and quality of the food offered.

*“I mean, I think something like this is a great idea… much more interested in the 57 existing stores like it’s, you know, this whole previous conversation is about new dollar stores, which is good to have. But we already have 57. I’d rather think through how they can provide, you know, more healthy food than just wanting to close them like thinking through how they could be an asset for the community rather than a detriment.” –* Policy Maker

Community members highlighted that if dollar stores were to add healthier items to the store, the pricing and the quality would need to be the same as other store types (like supermarkets) in order to be interested in purchasing them. Given the perceived lack of cleanliness at the store, and lack of staff to manage fresh items, community members felt that dollar stores would need to give extra attention to the quality, freshness, and expiration of those items. Regarding fresh meat, they had food safety concerns about handling and storing meat properly, again due to the lack of adequate staffing they have observed by many participants at their local dollar store.

*“It goes back to, are they going to keep the stores clean? Because, you know, with food you are going to have mice. And then it’s the city. In the city you breed rats you have to constantly keep the store clean, and a lot of times in these areas they do not keep the store clean.”* – Community Member

Participants were not unaware of the challenges the dollar stores might face in adding healthy, fresh items to their stores. They recognized the challenges described above with maintaining fresh items (quality, spoilage), and the refrigeration and equipment needs, which would cost money and take up space in the store. There were also concerns that the nature and structure of a corporation would be a barrier to negotiations around adding fresh food in a way that satisfies the consumer and the corporation. There were numerous strategies that community members and policy discussed in order to make a staple foods ordinance more feasible for dollar store corporations: see Table 4.

### Enforcement of dollar store policies

The issue of enforcement of the policies described above was a common theme throughout many of the interviews, and during both workshops. While there was high support for dollar store policies, there was also considerable concern about being able to enforce such policies and hold dollar stores accountable in the future.

#### Business licenses

Those that had experience with conditional use policies, including community members, did not feel that zoning code was the best option for dollar store policy due to enforcement difficulties. Zoning addresses the type of use, but fails to regulate the identity or business practices of the entities involved. Therefore, business licenses were suggested by policy makers as an alternative route, especially since they do not currently exist at the City level in Baltimore:

*“A license is able to get at the “Who?,” zoning normally deals with the “What?” …But licensing can get at the actual operator because it’s to an individual. If you misbehave, we can take the license away.”* – Policy Maker

Importantly, they emphasized that business licenses would be required for all businesses, not just dollar stores.

Participants also mentioned that by implementing business licenses, the City could enact formula business restrictions, meaning that they could decide to give out a limited number of business licenses for each business type. Thus, multiple dollar stores (or any store) would not be able to open so many of the same business model within a small geographic radius.

However, participants identified drawbacks to business licenses, including challenges in getting them “up and running” which requires staff time and capacity, and funding at the City level.

#### Enforcement at lease/permit renewal

An alternative strategy to introducing business licenses that was suggested involved tying policy requirements, such as community benefits agreements content and staple foods ordinances, to the building lease or business permit renewal process. By doing so, the City and the community could evaluate how well dollar stores are meeting the requirements outlined by such policies over time, and decide whether to renew their lease/permit. In order to accomplish this, the City would need to look at legislation around length of leases/permits, given that recently dollar stores in Baltimore City have recently been able to sign 10 year leases.

## Discussion

In this mixed-methods study of community member and policy maker support for dollar store policies, there was strong support for policies that would improve the dollar store environment for consumers (as opposed to shutting them down or banning them entirely), such as zoning board approval based on meeting certain requirements, a community benefits agreement, or a staple foods ordinance. For example, implementing a community benefits agreement or staple foods ordinance would allow new dollar stores to open, but would help better align their offerings with neighborhood priorities. Study findings were disseminated to Baltimore City Council in a written report, and to community members via email listservs and social media.

Previous studies of dollar stores conducted at the local level in DeKalb County Georgia, and New Orleans, Louisiana have been used to inform policy, but lack community perspectives on specific policy options (31, 32). Our findings do align with a recent nation-wide dollar store survey of 750 Americans conducted by CSPI, which explored consumer use and perceptions of dollar stores, and assessed how dollar stores could make healthy food more accessible (9). The survey found strong support for stocking and marketing of healthier foods at dollar stores, and an overarching sentiment that dollar stores have a responsibility to improve the health of the neighborhoods they serve (9). Given the lack of existing literature on community support for dollar store policies, future research should use community-engaged approaches, such as community member workshops like those described in the present study, to understand and generate policy recommendations at the local level.

Although there are multiple definitions of evidence-informed policy (33), they each often focus on scientific research as the evidence (34). However, sourcing evidence for policy should also include gathering local knowledge and interests, and applying that knowledge to designing policy (34). Health equity policy frameworks recognize that one of the root causes of inequities is an imbalance of power in decision making which may impact health outcomes (35). Creating and maintaining partnerships with community leaders and organizations and including them in the policy making process is critical for creating equitable policies that benefit the people they impact most (33, 35). In this study, local knowledge and priorities were incorporated into the policy development process. At the same time, policy maker perspectives were critical for understanding the feasibility of the dollar store policies being discussed by community members, particularly policies that would apply to existing dollar stores (as opposed to only addressing new ones opening). During the study period, participants generated one strategy for consideration to address existing dollar stores – businesses licenses. Alongside community-engaged approaches, further research and policy analysis is needed to identify additional feasible strategies, including speaking with policy makers in other locations who have successfully passed dollar store policies that address existing stores, as well as exploring how policies used outside of the retail space could be applied to dollar store issues.

Baltimore City currently does not issue business licenses (businesses are licensed at the Maryland State level), yet there was support for this strategy among both community members and policy makers. Participants were concerned about staff capacity at the city level required for implementation, and suggested tying the contents of a community benefits agreement to a store’s lease or building permit to allow for review at the time of renewal. This may require shortening of the allowable lease/permit length in Baltimore City and still begs the question of who will review each store’s compliance when up for lease/permit renewal.

There was strong support for a staple foods ordinance in Baltimore City among participants, as well as concern about enforcing such a policy if enacted. Given most dollar stores are SNAP-authorized retailers (6), they theoretically already meet minimum stocking standards for staple food items (the four staple food categories are: fruits or vegetables; meat, poultry or fish; dairy; and breads or cereals) (36). Minneapolis, Minnesota is the only municipality to date to pass a staple foods ordinance in 2015 which expands upon SNAP requirements to include additional standards for the types, varieties, and depth of stock of healthy foods (37, 38). The policy was enforced during the city’s standard enforcement procedures for health inspections: if the store was non-compliant after a series of re-inspections, their business license could be revoked (37). The evaluation study of this policy found significant improvements in healthy food availability in Minneapolis stores, which is promising given that dollar stores tend to offer a wide variety of ultra-processed foods and lack fresh produce (39–41). However, enforcement proved difficult due to limited staff capacity to conduct re-inspections for non-compliant stores, hampering the potential impact of the policy (37).

One strategy for encouraging dollar store compliance with a staple foods ordinance is to simultaneously increase demand for fresh, healthy foods. Participants in this study heavily emphasized produce, which is not required by SNAP or the Minneapolis ordinance. Dollar stores could participate in federal programs that offer fruit and vegetable incentives like SNAP healthy incentive programs (42) or the Gus Schumacher Nutrition Incentive Program (43), or become Special Supplemental Nutrition Program for Women, Infants, and Children (WIC) authorized retailers. There are also federal grants like the Healthy Food Financing Initiative that supports retailers in underserved areas to increase healthy food access, and local programs in Baltimore like Baltimore’s Fresh Crate program involving Loyola University and small business owners to bring fresh, local produce to the community (25). Importantly, lessons from previous interventions in other small food retail stores demonstrate store-level challenges with stocking fresh items related to refrigeration, spoilage issues, and lack of profitability/customer demand (44–47). Stores also need support in order to establish and maintain stock of fresh items, including technical assistance, support for infrastructure cost, and financial incentives in order to increase feasibility (45–48). Dollar stores are likely to face similar challenges with adding fresh produce to their stores which should be considered in future policies and interventions.

Other municipalities require community benefits agreements as a condition of land use approval. In 2019, Philadelphia, Pennsylvania passed an ordinance requiring, wherever feasible, a community benefits agreement between developers of High Impact Development Projects and host communities. While the city cannot mandate the contents of these agreements, the ordinance presents a list of suggested terms to include on a voluntary basis (49). Baltimore City could similarly outline suggested language for community benefits agreements related to healthy food stocking, store cleanliness and appearance, and proper staffing (as generated by study participants in Table 4). This creates a starting point for neighborhoods who are not aware of the potential impacts of dollar stores reported in other parts of the City, and/or who lack time, capacity, and resources to hold meetings and generate new ideas.

This study is not without limitations. While a community-engaged approach was used, the sample is subject to potential selection bias given the use of purposive, snowball, and convenience sampling. This could have, in theory, led to a sample with biased opinions of dollar stores. However, recruitment techniques such as distributing flyers in public spaces and recruiting in zip codes where there were little to no responses were used to capture a wide range of perspectives and experiences. Recruiting current and former dollar store staff in Baltimore City proved challenging; many staff said they were not allowed to speak to researchers and could not accept flyers. As a result, only one dollar store staff person from Baltimore City was included. Three former dollar store staff from outside of the city were recruited in an attempt to fill this gap. The sample size for the online survey was small due to receipt of numerous false responses despite careful avoidance of posting the survey link online or on social media where it could be shared widely among an ineligible audience. Security measures were implemented through Qualtrics to identify false responses and duplicate IP addresses, and additional criteria for inclusion was established based on the length of time to complete the survey, response patterns (i.e., “straight lining”, or selecting the same response for each question), accurate Baltimore City zip codes, and email address (50).

## Conclusion

This study is the first to assess community support for dollar store policies at the local level. Across a sequential mixed-methods approach, there was strong support for policies that improve dollar stores to align better with community priorities, as opposed to closing or banning dollar stores. Future research is needed to assess the implementation and evaluation of certain policies for dollar stores in Baltimore City and nationwide.

## Data availability statement

The raw data supporting the conclusions of this article will be made available by the authors, without undue reservation.

## Ethics statement

The studies involving humans were approved by Johns Hopkins University Institutional Review Board. The studies were conducted in accordance with the local legislation and institutional requirements. The participants written consent to participate in this study.

## Author contributions

SMS: Conceptualization, Investigation, Methodology, Project administration, Supervision, Visualization, Writing – original draft, Writing – review & editing. SRS: Investigation, Writing – review & editing. EL: Investigation, Writing – review & editing. SJ: Writing – review & editing. KG: Writing – review & editing. EF: Writing – review & editing. LP: Investigation, Project administration, Writing – review & editing. SH: Investigation, Writing – review & editing. SP-B: Conceptualization, Writing – review & editing. SB-N: Conceptualization, Writing – review & editing. JG: Conceptualization, Funding acquisition, Methodology, Project administration, Supervision, Visualization, Writing – review & editing.
