# Erratum: Dollar store policy opportunities in Baltimore City: community member and policy maker perspectives

**DOI:** 10.3389/fnut.2024.1509009

**Published:** 2024-10-24

**Authors:** Frontiers Production Office

**Affiliations:** Frontiers Media SA, Lausanne, Switzerland

**Keywords:** food access, food environment, community-informed policy, evidence-informed policy, Baltimore City, mixed-methods

Due to a production error, the following sentence was omitted from the **Funding Statement**: “Further support was provided to SM Sundermeir by the National Institute of Diabetes and Digestive and Kidney Diseases of the National Institutes of Health T32 Predoctoral Clinical Training Grant (T32DK062707).”

The correct Funding statement should read as follows:

